# Prevalence and whole genome sequencing analysis of Salmonella isolated from a homebred chicken farm in Jiangsu province

**DOI:** 10.3389/fvets.2026.1769606

**Published:** 2026-02-20

**Authors:** Ben Liu, Shaopeng Wu, Jiaqi Huang, Lulu Cui, Xiangbin Song, Wenli Tang, Shuhong Sun

**Affiliations:** 1 Shandong Provincial Key Laboratory of Zoonoses, College of Veterinary Medicine, Shandong Agricultural University, Tai'an, China; 2 Shandong Provincial Center for Animal Disease Control and Prevention, Shandong Provincial Center for Zoonoses Epidemiology Investigation and Surveillance, Jinan, China; 3 Shandong Center for Quality Control of Feed and Veterinary Drug, Shandong Provincial Key Laboratory of Quality Safety Monitoring for Animal Products and Veterinary Drug Innovation, Jinan, China

**Keywords:** antibiotic resistance genes, homebred chicken, Salmonella, sequence type, whole genome sequencing

## Abstract

*Salmonella* remains a significant zoonotic pathogen, and its antimicrobial resistance in agricultural settings poses a serious public health risk. In this study, we investigated the prevalence, serotype distribution, sequence types, and antimicrobial resistance profiles of Salmonella isolates from a homebred chicken farm in Jiangsu Province, China. Among 229 cloacal swab samples collected from two residential buildings, 60 *Salmonella* strains were isolated (isolation rate: 26.20%), with no isolates detected in environmental samples (*n* = 60). Differences in prevalence were observed among chicken breeds within the same building. All isolates belonged to two serotypes: *S*. Enteritidis (SE, 56.67%) and *S*. Kentucky (SK, 43.33%). Multilocus sequence typing (MLST) classified the strains into two sequence types: ST11 (*n* = 34) and ST198 (*n* = 26), with clear spatial clustering suggesting clonal dissemination within specific breeds. Antimicrobial susceptibility testing revealed high resistance rates to erythromycin (E), amoxicillin (AML), and ampicillin (AMP), exceeding 90%. All isolates were resistant to at least two antimicrobial agents, and one strain exhibited resistance to eight agents. Resistance gene screening showed that all isolates carried mutations in *gyrA* (S83F, D87G, D87Y) and *parC* (S80I). Additionally, *aph3'-Ib* and *aph(6)-Id* were detected in 68.33% of isolates. All strains harbored two or more antimicrobial resistance genes (ARGs). Whole-genome SNP analysis confirmed strong phylogenetic clustering by serotype and building, with ≤ 5 SNP differences within clades, indicating clonal persistence dissemination events. Homologous strain analysis (SNP distance ≤ 10) further revealed within-farm transmission of closely related strains. These findings highlight the clonal spread of multidrug-resistant *Salmonella* in chicken farms and underscore the need for improved surveillance and infection control measures in agricultural environments.

## Introduction

*Salmonella* is a food-borne zoonotic pathogen, which can be divided into more than 2,600 serotypes. The majority of these serotypes are zoonotic pathogens that infect humans and animals (1). The consequences of *Salmonella* infection in poultry include reduced productivity, increased mortality and elevated control costs, resulting in substantial economic losses for the poultry production industry (2). Reusable padding, water, feed, dust, infected people and contaminated equipment can be used as a repository for *Salmonella* in chicken houses (3). The horizontal transmission of pathogens among avian species in chickens can occur through contaminated food and water or via aerosols (4, 5). In some cases, *Salmonella* may become a permanent contaminant in the ventilation system, as it is difficult to clean and disinfect (2). The asymptomatic or symptomatic transmission of bacteria from infected animals can pollute the environment or directly infect others (6). For example, *Salmonella* pullorum is known to cause salmonellosis in poultry, resulting in significant economic losses for the poultry production industry. Healthy people can be infected with *Salmonella* by eating or handling contaminated, undercooked or raw chicken and products (7, 8). Furthermore, *Salmonella* has been identified as the primary bacterial cause of food-borne diseases on a global scale (9), thus posing a significant threat to food safety and public health (10). Consequently, effective measures to control *Salmonella* infection in poultry are imperative to minimize economic losses and human infections (11).

Antibiotics are widely used on poultry farms to promote growth and to prevent and treat bacterial infections. However, extensive and uncontrolled use of antibiotics can easily lead to the development of antibiotic-resistant bacteria (12). In addition, the development of drug resistance to key antimicrobial agents used in veterinary and human medicine poses a threat to the global healthcare system (13). In light of the mounting cognizance of AMR evolution in *Salmonella*, government entities and research organizations have initiated a longitudinal monitoring program, providing invaluable epidemiological data for risk assessment and the formulation of drug use guidelines (14).

Whole genome sequencing (WGS) has become a cost-effective and high-resolution genome analysis method, capable of providing significant information regarding antibiotic resistance genes (ARGs), genome mutation, MLST, and core genome MLST (cgMLST) (15–17). The World Health Organization has advocated for the implementation of genome-wide sequencing of *Salmonella*, to monitor the source and trend of transmission, and evaluate and monitor the effectiveness of prevention and control plans. The integration of WGS with epidemiological traceability data has been demonstrated to be a highly effective method for identifying the source of *Salmonella* infection. Furthermore, WGS has been demonstrated to facilitate the identification of determinants of AMR and the prediction of antibiotic resistance phenotype (18).

Despite the presence of homebred chicken farms in China, the epidemiology of *Salmonella* has received limited attention. To enhance comprehension of the dynamics of *Salmonella* in the context of homebred chicken farms, further study is required on the subject of *Salmonella* in chickens and the environment. The present study thus adopts a homebred chicken farm as its research object, employing epidemiological investigation and whole genome sequencing as its investigative tools. The primary objectives are to gain a comprehensive understanding of the epidemic situation and genetic relationship of *Salmonella* isolates and to elucidate the transmission law and epidemic characteristics of *Salmonella*.

## Materials and Methods

### Main reagents

Buffered peptone water (BPW), Tetrathionate broth (TTB)-enriched broth, Selenocysteine (SC)-enriched broth, Rappaport Vassiliadis *Salmonella* (RVS)-enriched broth, and Xylose-lysine deoxycholate (XLD) were purchased from HaiBo BioTech Ltd. (Qingdao, China).

### *Salmonella* isolates collection

In this study, *Salmonella* was isolated from 229 cloacal swab samples and 60 environmental samples collected from two chicken sheds in a homebred chicken farm in Jiangsu Province in August 2024. There are a total of 6 types of chickens raised in 2 chicken sheds buildings, with #202, #703 and #812 raised in Building No.1, and #802, #803 and #811 raised in Building No.2. Subsequently, the samples were aseptically transferred to a sample storage tube containing BPW. Following transportation to the laboratory at low temperature, each sample was added to 4.5 ml BPW and then incubated at 37 °C for 12 h for pre-concentration. Approximately 0.5 ml of the pre-enriched culture was then inoculated into 4.5 ml of TTB, SC and RVS, respectively. Cultures of each TTB, SC and RVS broth were then inoculated onto XLD agar medium and incubated at 37 °C for 48 h.

### DNA extraction and PCR

Genomic DNA was extracted from all *Salmonella* isolates using a commercial DNA kit (TIANGEN, Beijing, China). The quality and concentration of the bacterial genomic DNA were assessed by electrophoresis on a 1% agarose gel, and analysis was conducted using a NanoDrop2000 system (Thermo Scientific, Waltham, MA, USA) and a Qubit 4 fluorometer (Thermo Scientific, Waltham, MA, USA). The confirmation of smooth and round colonies without a black center or large colonies with a black center was achieved by polymerase chain reaction (PCR) assays with primers designed for the *FimW* gene (19).

### Antimicrobial susceptibility testing

All *Salmonella* strains were assessed for resistance to 12 antimicrobial drugs' susceptibility via the disk diffusion method, according to the Clinical and Laboratory Standards Institute (CLSI) (20). Briefly, the suspensions of bacterial were cultured and adjusted to the 0.5 McFarland turbidity standard, then using cotton swab to streak over the entire surface of Mueller–Hinton agar (Qingdao Hope Bio-Technology Co., Ltd., Qingdao, China). Then, the antibiotic susceptibility test paper were affixed to the plates and incubated at 37 °C for 16–18 h. Moreover, the diameter of the inhibition zone was measured with a ruler. The 12 antibiotics tested were ampicillin (AMP, 10 μg), amoxicillin (AML,20μg), trimethoprim-sulfamethoxazole (SXT, 25μg), tetracycline (TET, 30 μg), streptomycin (S, 10 μg), erythromycin (E, 15 μg), doxycycline (DOX, 30 μg), florfenicol (FFC, 30 μg), ofloxacin (OFX, 5 μg), gatifloxacin (GAT, 5 μg), ceftazidime (CAZ, 30 μg), cefoxitin (FOX, 30 μg). *Salmonella* strains resistant to more than three classes of antimicrobials were defined as multidrug-resistance (MDR) strains (21).

### WGS

All the strains were tested using second-generation high-throughput sequencing technology based on the Illumina HiSeq nova 6,000 platform with 150 bp paired-end reads (Novogene Co. Ltd, Tianjin, China). The original data of the WGS were spliced by quality control (https://enterobase.warwick.ac.uk/) and then uploaded to the website of Center for Genomic Epidemiology (CGE) for partial bioinformatics analysis (22). Among them, the tool (https://cge.food.dtu.dk/services/SeqSero/) was used to analyze the serotype of *Salmonella*.

### Gene annotation and analysis

The assemblies were serotyped by SISTER-cmd v1.1.1 (24) and then screened against the *Salmonella* seven-locus multi-locus ST database (MLST Github https://github.com/tseemann/mlst), ResFinder 4.1 tool (https://cge.food.dtu.dk/services/ResFinder/) (23), Resfinder database (25), PlasmidFinder Database (26), Pointfinder database (27) using Staramr v.2.0.1 (28) to assign STs and detect acquired ARGs, plasmid replicons and point mutations. The results were transformed into a binary table in R v.3.6.0 to analyse the presence/absence of acquired ARG alleles and the prevalence of each gene in different isolates grouped by serotypes or background information. All assemblies were annotated with Prokka v.1.14.6 (29) and subjected to a pangenome analysis with Roary v.3.12.0 (30).

### Phylogenetic analysis based on core-gene alignment and cgMLST

The full set of 60 genomes was used to generate a core-genome alignment and construct a maximum likelihood phylogenetic tree, using FastTree v.2.1.10 with gtr model. The midpoint rooted phylogenetic tree was annotated in by ggtree and ggtreeExtra. Minimum spanning trees were created based on cgSTs using GrapeTree v1.5.153 (31) with MSTree V2 algorithm. The cgMLST allele profiles were annotated using cgMLST.py v1.2.0 with 3,002 allele genes. Genomic diversity of the isolates within each source was assessed by calculating all pairwise allelic differences (PADs) between any two isolates. When multiple isolates shared the same cgST, one isolate was randomly picked and used for PAD calculation to ameliorate potential sampling biases caused by the overrepresentation of closely related isolates. To obtain more accurate evidence of *Salmonella* transmission, we further calculated the SNP distance using core-gene alignment (core genome size ~3.1Mb) of 60 genomes with SNP-dists v0.7.0 (https://github.com/tseemann/snp-dists). Isolate combos differed by ≤ 10 SNPs and were considered as potential transmission cases. Transmission cases are visualized by ggplot2 within R v.3.6.0.3.

## Results

### Isolation of *Salmonella* strains

**Figure 1:**
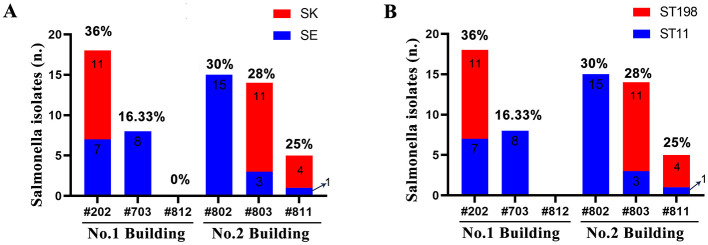
Prevalence of serotypes and STs in Salmonella strains. **(A)** serotypes. **(B)** STs.

Through systematic analysis of 292 samples from two buildings in a chicken farm, 60 strains of *Salmonella* isolates were observed in cloacal swabs, with a positive rate of 26.20% (60/229). However, *Salmonella* was not isolated from 60 environmental swabs. Further analysis shows that there are differences in the prevalence of *Salmonella* among different chicken breeds in residential buildings. In Building 1, the positive rates of *Salmonella* in three chicken breeds were 36% (18/50), 16.33% (8/49), and 0% (0/10), respectively (Figure 1A). In Building 2, the positive rates of *Salmonella* in the three chicken breeds were 30% (15/50), 28% (14/50), and 25% (5/20), respectively (Figure 1A).

### Serotype detection

Serotyping analysis classified the 60 *Salmonella* isolates into two serotypes: SE (56.67%, 34/60) and SK (43.33%, 26/60). Spatial distribution patterns revealed exclusive SE dominance in breeds #703 and #802 (Figure 1A), suggesting potential serovar-specific ecological niche adaptation within the environment.

### STs detection

Phylogenetic analysis of MLST revealed distinct strain stratification among the 60 *Salmonella* isolates, with all strains clustering exclusively within two STs: ST11 (*n* = 34, 56.67%) and ST198 (*n* = 26, 43.33%) (Figure 1B). Notably, spatial clustering patterns demonstrated complete ST11 dominance in breeds #703 and #802, suggesting clonal dissemination of specific lineages within the farm ecosystem.

### Antimicrobial susceptibility testing results

**Figure 2:**
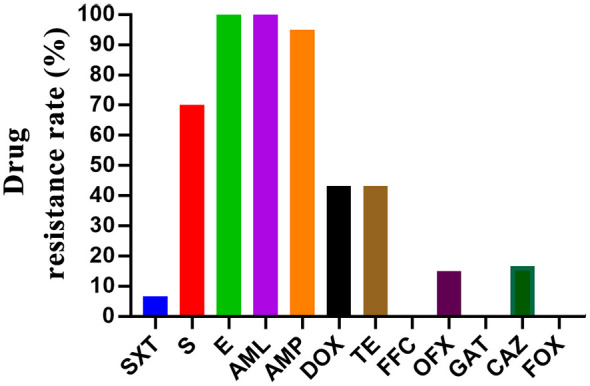
Analysis of antibiotic resistance in Salmonella strains. Rates of antibiotic resistance among Salmonella strains.

Following the genetic characterization of the isolates, we further evaluated their phenotypic antimicrobial resistance profiles to assess the potential public health risks associated with these prevalent strains. Antimicrobial susceptibility profiling of the 60 *Salmonella* isolates revealed divergent resistance patterns across 12 antimicrobial agents (Figure 2, Supplementary Table S1). It was observed that the resistance rates to E, AML, and AMP were all above 90% in all the *Salmonella* strains. In addition, all the tested *Salmonella* strains were resistant to at least 2 antibiotics (Table 1). A total of eight types of resistance profiles were identified among the strains, with E-AML-AMP and S-E-AML-AMP being the most prevalent (25%), followed by S-E-AML-AMO-DOX-TE (13.33%) (Table 1). Notably, one of these strains was resistant to 8 kinds of the tested antibiotic categories, with the resistance profile of S-E-AML-AMP-DOX-TE-OFX-CAZ (Table 1).

**Table 1:** The drug resistance profiles and proportion of Salmonella strains.

**Type of drug resistance**	**Drug resistance profile**	**Proportion**	**Total**
2	E-AML	1.67% (1/60)	1.67% (1/60)
3	E-AML-AMP	25% (15/60)	25% (15/60)
4	S-E-AML-AMP	25% (15/60)	26.67% (16/60)
	SXT-E-AML-AMP	1.67% (1/60)	
5	S-E-AML-DOX-TE	3.33% (2/60)	8.33% (5/60)
	S-E-AML-AMP-DOX	1.67% (1/60)	
	S-E-AML-AMP-TE	1.67% (1/60)	
	E-AML-AMP-OFX-CAZ	1.67% (1/60)	
6	S-E-AML-AMO-DOX-TE	13.33% (8/60)	15% (9/60)
	SXT-S-E-AML-AMO-TE	1.67% (1/60)	
7	S-E-AML-AMO-DOX-TE-CAZ	6.67% (4/60)	20% (12/60)
	SXT-S-E-AML-AMP-DOX-TE	3.33% (2/60)	
	S-E-AML-AMP-DOX-TE-OFX	10% (6/60)	
8	S-E-AML-AMP-DOX-TE-OFX-CAZ	3.33% (2/60)	3.33% (2/60)

### Detection of ARGs

**Figure 3:**
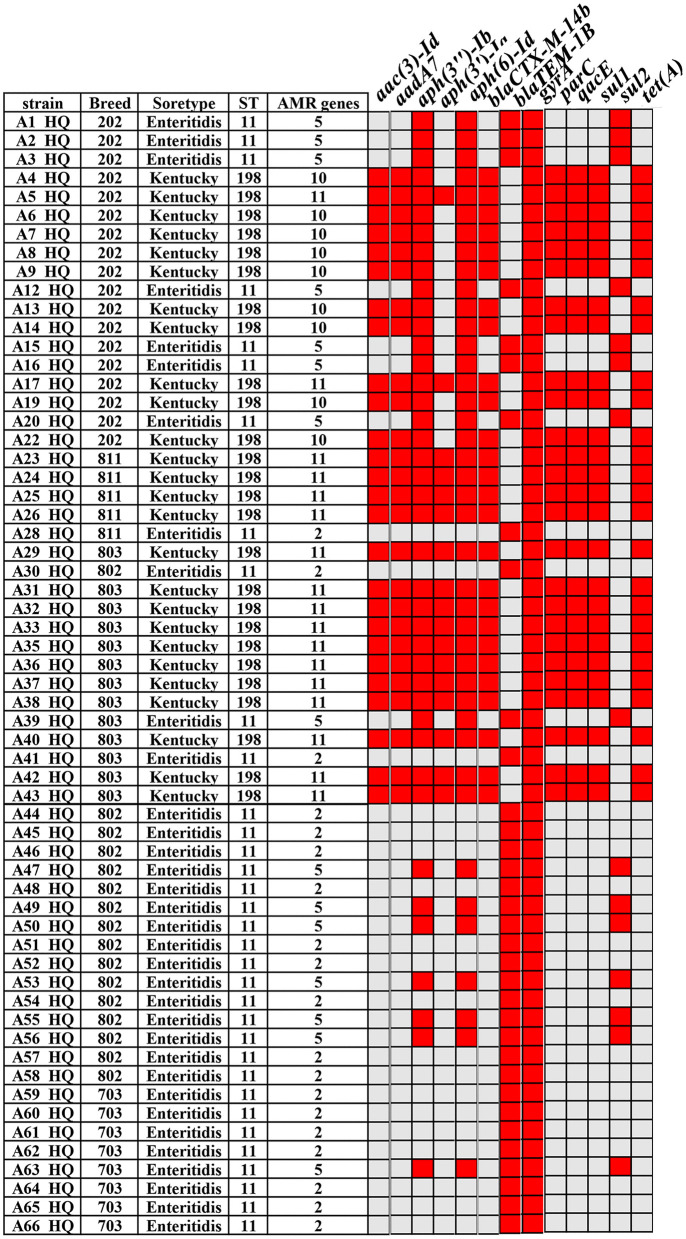
Specific information of Salmonella strains, including serial number, breed, STs, serotype, ARG number and drug resistance gene. The red dot means present resistance gene.

**Figure 4:**
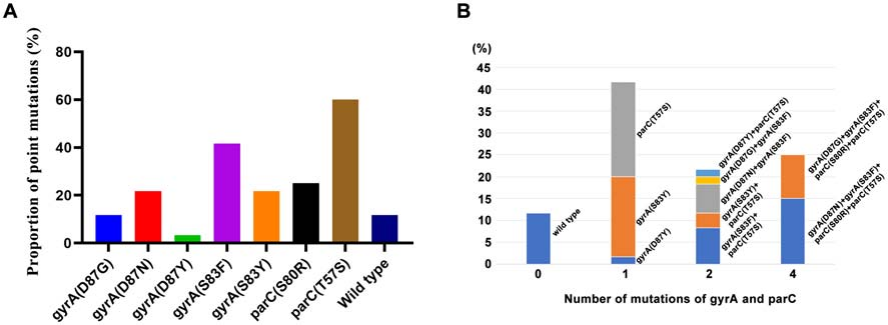
Frequency of mutations of ARGs (gyrA and parC) in Salmonella strains. **(A)** proportion of single site mutations. **(B)** proportion of multiple site mutations.

To further elucidate the genetic determinants underlying the observed multidrug-resistant phenotypes, we subsequently detected and analyzed the prevalence of ARGs in these isolates. The specific ARG information of *Salmonella* strain is shown in Figure 3. Firstly, the most common ARGs are *gyrA* [100% (60/60)], followed by *aph(3”)-Ib* and *aph(6)-Id*, with the carrying rates of 68.33% (41/60). In addition, the proportion of *Salmonella* carrying two ARGs was 31.67% (19/60), while the proportion of *Salmonella* containing 5, 10 and 11 ARGs was 25% (15/60), 15% (9/60) and 28.33% (17/60), respectively (Figure 3). Overall, the proportion of *Salmonella* carrying two or more ARGs reached 100%. ADDIN EN.CITE (32–35). As the *gyrA* gene is present in all Salmonella strains, we analyze the mutation analysis of the ARGs including *gyrA* and revealed significant mutations at several key sites: *parC* (T57S; 60%), *gyrA* (S83F; 41.67%), *gyrA* (D87Y; 56.67%), *gyrA* (S83F; 43.33%), and strain without SNP mutation sites of *gyrA* or *parC* were found in 7 strains of *Salmonella* (Figure 4A). Further analysis of the mutations showed that 21.67% of the strains carried two mutations, and 25% of the strains carried four mutations at the same time (Figure 4B), of which *gyrA* (D87N)+ *gyrA* (S83F)+ *parC* (S80R)+ *parC* (T57S) accounted for 15% at most.

### Genome-wide SNP analysis of *Salmonella* strains

**Figure 5:**
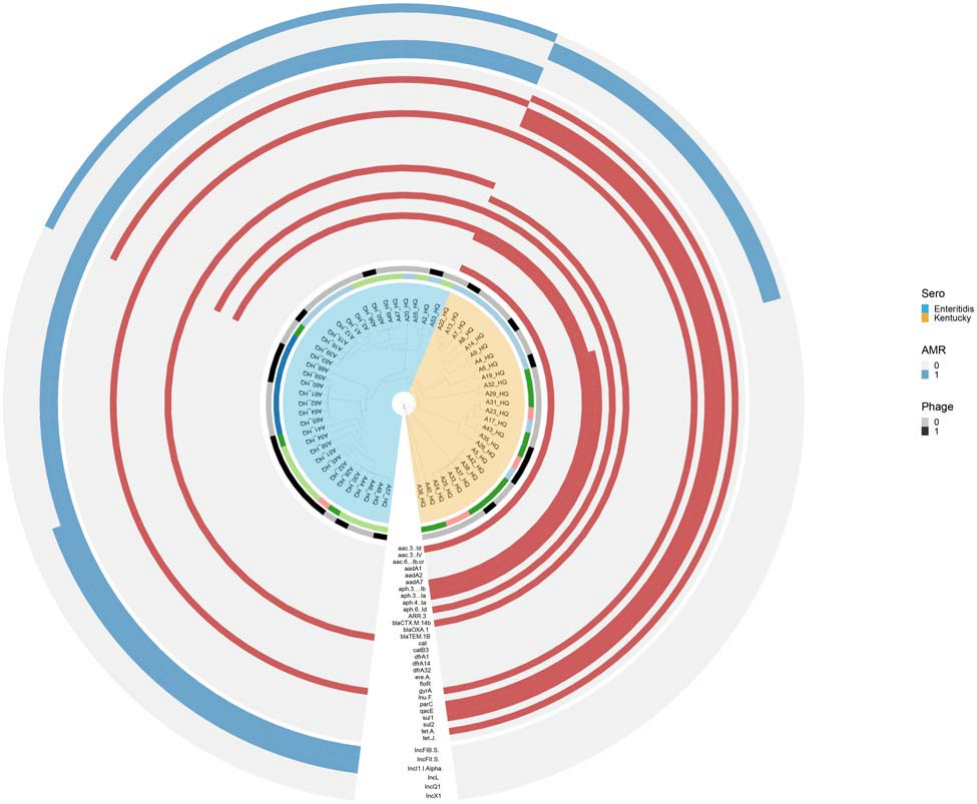
The phylogenomic relationships among 60 Salmonella strains. Different STs, serotypes, breeds, and antimicrobial resistance genes are indicated in various colors. The first circle represents the serotypes of isolated Salmonella strains, the second circle represents the breeds of isolated Salmonella strains, the third circle represents the ARGs of the strains, the fourth circle represents the plasmid of strains, from the inside to the outside.

Genome-wide SNP analysis was used to investigate the deep phylogenetic relationship among 60 *Salmonella* strains (Figure 5). The analysis showed that strains sharing identical serovar designation (SE/SK) formed discrete monophyletic clades (bootstrap support >90%), with ≤ 5 SNP differences within clades. In addition, isolates from the same building clustered, showing ≤ 3 SNP variations. Moreover, it can be seen that *Salmonella* belonging to the same serotype may come from different buildings, demonstrating potential inter-unit transmission events.

### Homologous strain analysis

**Figure 6:**
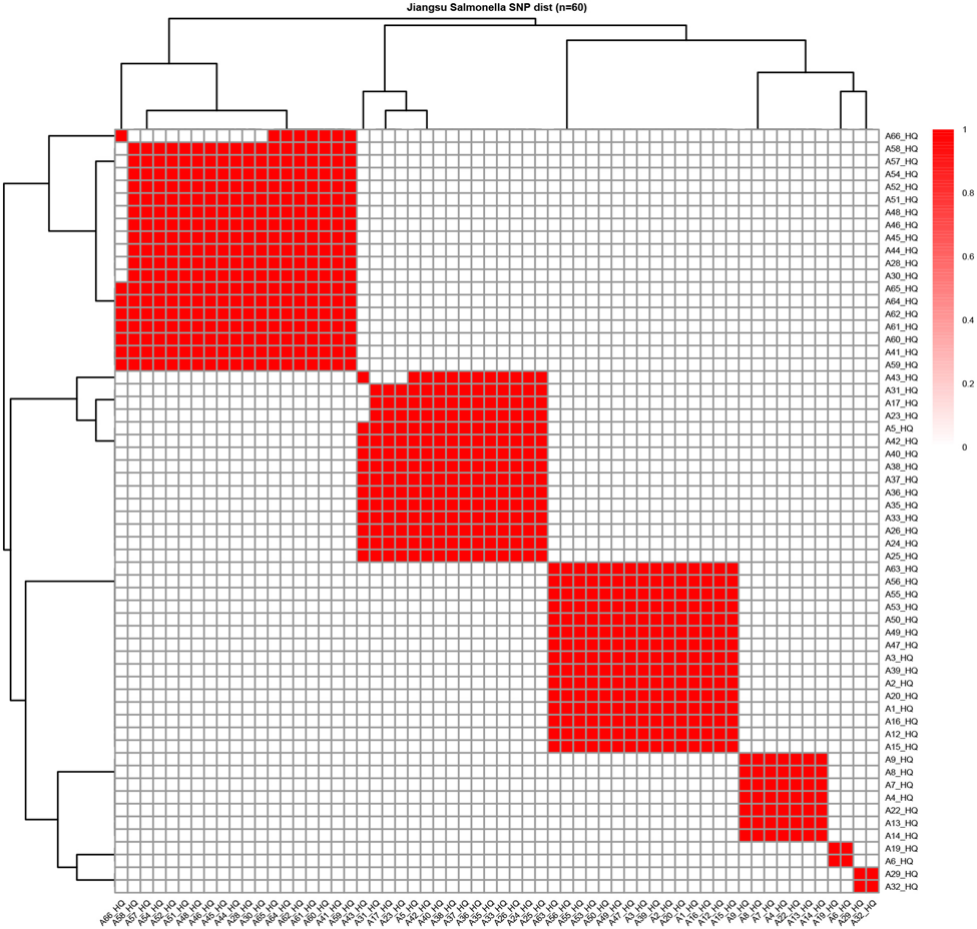
Heat map of homologous salmonella. After extracting the core genomes of these 60 strains of Salmonella from Jiangsu, the multi-sequence alignment was carried out, and then the SNP distance between two strains was calculated. Two strains with SNP ≤ 10 were defined as homologous strains, and the heat maps of these homologous strains were drawn. The red ones indicated that the two strains were homologous.

Following core genome extraction from 60 *Salmonella* strains isolated in Jiangsu Province, we performed multiple sequence alignment and calculated single nucleotide polymorphism (SNP) distances between strain pairs. Homologous relationships were defined using a stringent current criterion (SNP distance ≤ 10), representing only 10 nucleotide differences across the 3,000,000 bp core genome. The constructed heatmap highlights homologous pairs in red, revealing that the *Salmonella* isolated from different species were closely related, indicating that some *Salmonella* had strong transmission ability and could spread across environment (Figure 6).

## Discussion

The extensive and inappropriate utilization of antimicrobial agents has expedited the emergence and propagation of AMR (36). While AMR *Salmonella* infection has been reported on a global scale, the number of cases in developing countries is increasing at an alarming rate (37). The clinical use of antibiotics is a primary method of treating *Salmonella* infections. Chickens serve as natural *Salmonella* reservoirs, enabling zoonotic transmission through food chains. *Salmonella* infection in chickens and chicken farms is a significant concern and homebred chicken farms may play a significant role in the transmission of human salmonellosis (38). Therefore, it is necessary to detect *Salmonella* regularly in homebred chicken farms. Strict management measures, such as environmental disinfection, are effective in reducing the prevalence of *Salmonella* during the breeding and hatching processes.

In this study, a chicken farm in Jiangsu Province, where 229 cloacal swabs and 60 environmental swabs were collected in August 2024, and 60 strains of *Salmonella* were isolated and identified. Using WGS technology facilitates precise identification of the serotype of *Salmonella*. The predominant serotypes identified in this study is *S*. Enteritidis and *S*. Kentucky. As one of the most prevalent serotypes of non-typhoid *Salmonella* infection, SE in shelled eggs is widely regarded as the causative agent of large-scale foodborne outbreaks (39, 40). As previously reported, the average detection rate of SE in China was 1.5% from 2006 to 2012 and 15.1% from 2013 to 2021 (41). In this study, SE was identified as the most prevalent serotype, a finding that aligns with earlier research conducted in China (38, 41). The MLST model based on WGS showed that there were two kinds of ST in 60 *Salmonella* strains. The most prevalent STs were found to be ST11, followed by ST198, which is consistent with the findings of previous studies. Furthermore, the SE isolated from eggs and chickens also belongs to only one sequence type, namely ST11 (42, 43). Consequently, ST11 has been identified as the predominant sequence type, accounting for approximately 90% of the EnteroBase database of SE worldwide, which underscores the interconnectedness of poultry-associated AMR risks across continents (44).

For an extended period, the primary approach to disease prevention and control in China has relied on the utilization of antibiotics. This strategy has contributed to the high prevalence of drug-resistant bacteria and has presented significant challenges to prevention efforts. This study examined the antibiotic resistance patterns of isolates, with all isolates exhibiting resistance to a minimum of two different antibiotics. The isolates demonstrated high resistance levels to macrolides and penicillin, followed by compound glycosides. The resistance rate to erythromycin is more than 95%, which is similar to the previous research results (45, 46). This suggests that resistance to erythromycin is likely an intrinsic property of Salmonella, precluding its use for treating salmonellosis in chickens. These trends align with WHO reports of >50% AMR *Salmonella* in low/middle-income countries' poultry sectors. This phenomenon may be attributed to the predominance of AMP as the primary treatment for *Salmonella*, accompanied by an escalating trend in antibiotic resistance, which ultimately leads to the development of high-level drug resistance. The present study demonstrates the antibiotic resistance phenotypes were largely consistent with homologous ARGs. In this study, 29 types of ARGs were identified, with 11 types accounting for the highest percentage of 28.33%. The results showed that SK carried more ARGs than SE. In recent years, SK has become a common NTS associated with human infection (32, 33). SK is resistant to a variety of antibiotics (including aminoglycosides, beta lactams, sulfamethoxazole and tetracycline), and carries a variety of resistance genes such as *aac(3)-Id, aada7, bla*_*TEM*−1_, *sul1* and *tet(A)* (34, 35). Some isolates subsequently became highly resistant by obtaining resistance genes encoding extended spectrum *bla*_*CTX*−*M*_, cephalosporinase (CMY) or carbapenem lactase (oxa-48, vim and NDM). These genes are often associated with IncC and IncI1 plasmids. Furthermore, the most prevalent mutations of ARGs are *gyrA* and *parC*. Mutation analysis demonstrated that *parC* (T57S) and *gyrA* (S83F) exhibited the highest mutation frequency, while prior reports substantiated that *parC* (T57S) was the most prevalent mutation in quinone resistance-determining regions (QRDR) (47). In addition, strains exhibiting mutations in both *gyrA* (S83F) and *parC* (T57S and S80I) demonstrated a high level of fluoroquinolone (FQ) resistance (48). Notably, resistance to quinolones and fluoroquinolones, particularly ciprofloxacin resistance, is a growing problem (49). Moreover, this study also found that 25% of the strains carried four mutations at the same time, including *gyrA* (D87N)+*gyrA* (S83F)+*parC* (S80R)+*parC* (T57S) and *gyrA* (D87G)+*gyrA* (S83F)+*parC* (S80R)+*parC* (T57S) (Figure 4B).

Despite the clear evidence of Salmonella infection in clinically affected chicken, both environmental swabs and cloacal swabs from asymptomatic contacts yielded negative results. This discrepancy may be attributed to several factors. Firstly, the implementation of stringent and routine biosecurity measures, such as frequent disinfection of facilities, equipment, and feeders, could effectively reduce the bacterial load in the environment to levels below the detection limit of culture methods. Secondly, inherent limitations of the sampling methodology, including the selection of specific sampling sites, the volume of material collected, and the potentially low and patchy distribution of pathogens in the environment, might have contributed to a false-negative outcome. Furthermore, the timing of sampling is critical; it is plausible that extensive sanitation procedures were initiated following the initial disease outbreak, thereby temporarily eliminating environmental contamination. Similarly, the negative results from all cloacal swabs of asymptomatic chicken suggest that, at the time of sampling, the infection may have been relatively contained within the clinically ill cohort and had not yet established widespread subclinical carriage or shedding within the broader flock. This pattern could reflect the early stage of the outbreak or the effectiveness of initial control measures in limiting horizontal transmission. While these negative results prevent us from mapping a complete transmission chain, they are nonetheless informative. They highlight the challenges in detecting intermittent or low-level shedding and environmental contamination, and underscore the critical importance of robust and persistent biosecurity protocols in breaking the cycle of infection, even when pathogens are not readily detectable in the immediate environment.

While this study provides comprehensive genomic insights, several constraints should be considered when interpreting the results. The exclusive focus on a homebred chicken farm from Jiangsu Province, while providing detailed regional data, limits the generalizability of findings to other regions with different agricultural practices. Moreover, this study is a cross-sectional survey that reveals the possibility of transmission, but cannot prove dynamic transmission events.

## Conclusion

In summary, 60 strains of *Salmonella* were collected from 229 cloacal swabs and 60 environmental swabs from a chicken farm in a homebred chicken farm in Jiangsu Province in August 2024. The main serotypes are *S*. Enteritidis and *S*. Kentucky, and ST11 and ST198 are the corresponding ST types respectively. The potential risk of food-borne infection caused by AMR *Salmonella* is emphasized, and we should pay close and continuous attention to *Salmonella* isolated from animal-derived foods. Our findings provide baseline data for priority interventions to ensure food safety and public health.

## Data Availability

The original contributions presented in the study are included in the article/Supplementary material, further inquiries can be directed to the corresponding authors.

## Supplementary material

Supplementary Table 1 Detailed information on the drug-resistant phenotype of Salmonella strains.
